# PERINATAL CARE IN A NORTHEASTERN BRAZILIAN STATE: STRUCTURE, WORK PROCESSES, AND EVALUATION OF THE COMPONENTS OF ESSENTIAL NEWBORN CARE

**DOI:** 10.1590/1984-0462/;2019;37;2;00003

**Published:** 2019-02-25

**Authors:** Felipa Daiana Bezerra, Maria Alexsandra da Silva Menezes, Rosemar Barbosa Mendes, José Marcos de Jesus Santos, Débora Cristina Fontes Leite, Samir Buainain Kassar, Ricardo Queiroz Gurgel

**Affiliations:** a Universidade Federal de Sergipe, Aracaju, SE, Brazil.; b Universidade Federal de Sergipe, Lagarto, SE, Brazil.; c Universidade Tiradentes, Aracaju, SE, Brazil.; d Universidade Estadual Ciências da Saúde de Alagoas, Maceió, AL, Brazil.

**Keywords:** Perinatal care, Infant, newborn, Child health services, Intensive care, neonatal, Assistência perinatal, Recém-nascido, Serviços de saúde da criança, Terapia intensiva neonatal

## Abstract

**Objective:**

To describe the structure and the processes of care for pregnant women/newborn infants, including the Essential Newborn Care (ENC), in maternity hospitals in Sergipe State, Brazil.

**Methods:**

A cross-sectional study carried out between June 2015 and April 2016 in all maternity hospitals of Sergipe with more than 500 deliveries/year (n=11). A questionnaire on the existing structure and work processes was administered to the managers. Subsequently, a representative number of postpartum women from these hospitals were interviewed (n=768). Their medical records, as well as newborn infants’ records, were also analyzed.

**Results:**

Sergipe has 78 beds of Neonatal Intensive Care Unit (NICU) and 90 beds of Intermediate Care Unit (IMCU) to meet spontaneous and programmed demand. Only six maternity hospitals (54.5%) performed the risk classification, and four (36.3%) had protocols for high-risk parturient care. Regarding the ENC components, only 41% (n=315) of the women had early skin-to-skin contact with their babies, 33.1% (n=254) breastfed in the first hour of life, and 18% (n=138) had a companion always during birth.

**Conclusions:**

The distribution of NICU beds between capital city and other cities of the State is adequate, considering Brazilian guidelines. However, there was a low adherence to the protocols for hypertensive and hemorrhagic emergencies, and a low coverage of humanization policies, pregnancy risk classification and ENC practices, especially breastfeeding in the first hour of life, and companion always during birth.

## RESUMO

**Objetivo:**

Descrever a estrutura e os processos de atendimento a gestante/recém-nascido, incluindo os componentes do *Essential Newborn Care* (ENC), das maternidades de Sergipe, Brasil.

**Métodos:**

Estudo transversal realizado entre junho de 2015 e abril de 2016 em todas as maternidades de Sergipe (n=11) com mais de 500 partos/ano. Foi aplicado um questionário aos gestores sobre a estrutura e os processos de trabalhos existentes. Posteriormente, um número representativo de puérperas desses hospitais foi entrevistado (n=768) e seus prontuários, bem como o dos recém-nascidos, foram analisados.

**Resultados:**

Sergipe conta com 78 leitos de Unidade de Terapia Intensiva Neonatal (UTIN) e 90 de Unidade Intermediária (UI) para atendimento da demanda espontânea e programada. Somente seis maternidades (54,5%) realizam a classificação de risco e quatro (36,3%) possuem protocolos para atendimento das parturientes de alto risco. No que se refere aos componentes do ENC, apenas 41% (n=315) das mulheres tiveram contato pele a pele precoce com seu filho, 33,1% (n=254) amamentaram na primeira hora de vida e 18% (n=138) tiveram a presença do acompanhante em todos os momentos do parto.

**Conclusões:**

A distribuição de leitos de UTIN entre capital/interior é adequada no Estado, levando-se em consideração a legislação vigente no país. Entretanto, houve baixa adesão aos protocolos das emergências hipertensivas e hemorrágicas, e baixa cobertura das políticas de humanização, da classificação de risco para a gestante e das práticas do ENC, principalmente quanto à amamentação na primeira hora de vida e à presença do acompanhante na parturição.

## INTRODUCTION

The organization of perinatal care, based on a risk approach, implies that every pregnant women and newborn infant receives adequate care at the level of complexity they need. ^1^ Therefore,the existence of a structure is essential in order to provide specialized medical support, diagnostic and therapeutic procedures necessary in each case, as well as the follow-up protocols based on the best available scientific evidence.^2^

In Brazil, although the World Health Organization’s (WHO) latest report on the maternal mortality ratio (MMR) has shown a significant reduction in the number of maternal deaths from 104 in 1990 to 44 in 2015 per 100,000 live births,^3^ the country has not reached the fifth Millennium Development Goal (MDG) target yet, which recommended a reduction of – in the MMR by 2015.^4^ In addition, a national survey, with similar methodology to the one used in the WHO report, showed an even worse situation in the Northeast region, with emphasis on the state of Sergipe, whose estimate of MMR was 90.1 maternal deaths per 100,000 live births.^5^

As for neonatal mortality, the main component of infant mortality since the 1990s, Lansky et al.^6^ found a mortality rate of 11.1 neonatal deaths per thousand live births in the country and, similarly, the Northeastern region has a higher concentration of these deaths. Therefore, it is evident that there is a need to adapt health care practices and maternal and child health services to improve these health indicators in the region.

The existence of an effectively regionalized and hierarchical system of perinatal care is not yet a reality in Brazil. Althoughsome progress has occurred and contributed to the reduction of neonatal and maternal mortality, this has not happened uniformly at national level.^7^ Public policies try to reverse this scenario and organize the perinatal health in the country, highlighting actions of the Brazilian Ministry of Health still in the 1990s, as the Program to Support the Implementation of the State Hospital Referral Systems for High-Risk Pregnancy Care and the Prenatal and Birth Humanization Program (PBHP).^8^ More recently, the *Rede Cegonha* (Stork Network), a maternal care program, has been created. This strategy proposes changes in the model of birth care with multiprofessional healthcare team, use of protocols, and surveillance of health indicators.^9^

However, labor and birth care services still present low quality, such as inadequate follow-up for the labor process, associated with no use of simple and effective technologies, due to the lack of care protocols or due simply to their non-compliance by healthcare providers. There is also a lack of medication, equipment for diagnosis, availability and preparation of the team.^10^

Considering that saving mothers and newborns is rarely the result of a single intervention, but of a complex network of interlocking actions,^11^ it is important to consider the international document Essential Newborn Care (ENC). This document presents a relatively simple set of practices which may reduce neonatal mortality.^12^

Given the perinatal health problems in Brazil, especially in the North and Northeast regions, associated with the fact thatthe state of Sergipe does not have studies with this approach and magnitude, the present study aimed to evaluate the characteristics of neonatal care in Sergipe from the appreciation ofits structure and work processes, including the evaluation of ENC practices in maternity hospitals in the state.

## METHOD

A cross-sectional study was carried out between June of 2015 and April of 2016 in Sergipe, a state of the Northeast region of Brazil.

Initially, data from maternity hospitals performing more than 500 births per year in Sergipe were collected by a questionnaire evaluating the structure of the neonatal units, as well as their work processes. The questionnaires were filled in during in-person interviews with the managers of the selected establishments. All 11 maternity hospitals working during the study period were included.

Four domains were evaluated:

- Infrastructure: availability of Neonatal Intensive Care Unit (NICU) and Intermediate Care Unit (IMCU) beds, geographical location of the maternity hospitals and neonatal intensive care beds, presence of support units such as blood bank or transfusion agency, access to human milk, availability of ambulance for transportation, and existing work processes (adherence to the protocols for eclampsia, pre-eclampsia and hemorrhage, presence of risk classification in the maternity hospitals, humanization policies).
- Human resources profile: existence of medical and nursing coordinators with specialization in obstetrics or neonatology and presence of a pediatrician in the birth room during 24 hours uninterrupted.
- Available medications: beta blockers, methyldopa, hydralazine, nifedipine, corticosteroids, misoprostol, uterine contraction inhibitors, magnesium sulfate, antihemorrhagic drugs, silver nitrate, anti-D immunoglobulin and surfactant.
- Equipment: resuscitation materials for both mother and newborn (laryngoscope, self-inflating manual ventilation bag, mechanical ventilator, suction probes) and radiant heat source for newborns.

The questionnaire used in this study was retrieved from a previous publication,^13^and it was administered by only one interviewer to the managers of the units included.

In a second moment, postpartum women were interviewed and had their medical records and their newborns’ records analyzed after their discharge. A representative sample size of 768subjects was obtained. The post-partum women were selected by a simple random sampling from a list of daily admission. All women with live fetuses and stillbirths with birth weight greater or equal to 500 g and/or gestational age greater or equal to 22 weeks were considered eligible. Allocation was proportional to the size of the institution. Women who did not speak Portuguese, who presented some type of physical or mental incapacity to answer the questions, who had their baby born in transit or who refused to participate in the study were not included.

In order to carry out the interviews with the post-partum women, the researchers remained at least seven days in each institution. If the number of puerperal women was reached before this period, a random lottery would be drawn up with the limitation of interviewees’ daily number, wherefore the seven days were reached. Interviews happened at least six hours after delivery. The prenatal cards were photographed, and the information entered into the database.

ENC is a set of practices that help to reduce neonatal mortality.^12^ The investigated items were: number of prenatal consultations, orientation on the reference maternity hospital for birth, use of antenatal corticosteroids in cases of preterm birth risk between 26 and 34 weeks, use of partograph, presence of a companion in all the moments of childbirth, early skin-to-skin contact, and breastfeeding in the first hour of life.

For the statistical analysis, univariate and bivariate techniques were used to obtain the distribution of frequency and percentage values. Pearson’s chi-square test of independence was used to investigate associations between categorical variables with significance level of p<0.05. Fisher’sexact test was used for the categories with low frequency cells, considering the same level of significance. It was estimated the *Odds Ratio* (OR) as a measure of association and their respective 95% confidence intervals (95%CI) using the Mantel-Haenszel method. The analysis was conducted in Statistical Package for the Social Sciences (SPSS) 20.0 Mac (SPSS Inc., Chicago, IL, USA).

The study was approved by the Research Ethics Committee of the Federal University of Sergipe, opinion nº 453.279/2013, CAAE 22488213.4.0000.5546. All care was taken to ensure confidentiality of the information according to Resolution nº 196/96 of the National Health Council of the Brazil’s Ministry of Health. The postpartum women signed the consent term with guarantee of refusal at any time, without suffering any damage.

## RESULTS

The 768 postpartum women and the 11 maternity hospitals eligible to participate in the study were analyzed. There was no loss or dropout during the study.

A total of 78 NICU and 90 IMCU beds were identified in service in Sergipe. Most of these beds were in the state’s capital city (Aracaju) (NICU: 100.0%, IMCU: 75.5%) and similarly distributed between public and private services (NICU: 43.6 x 56.4%, IMCU: 52.2 x 47.8%). Table 1 shows the distribution of NICU and IMCU beds in the state of Sergipe according to the geographical location (capital city and town) and type of financing (public, private and mixed), in addition to information about structure and work processes in the eligible maternity hospitals.

**Table 1:** Proportional distribution of factors related to the structure, work processes and other characteristic of public, private and mixed maternity hospital in the state (n=11). Sergipe, Brazil, 2015-2016.

Maternity hospitals (Financing - Location)	Structure							Work processes		
	NICU beds*		IMCU beds**		Pediatrician on call 24h	Resuscitation equipment	Availability of required medications	Adherence to risk classification	Existence of ≥1 protocol	Team adherence to the 4 protocols
	n	%	n	%	%	%	%	%	%	%
A (Public - Capital)	34	43.6	25	27.8	100	100	92	100	100	50***
B (Mixed - Capital)	30	38.5	30	33.3	100	100	100	100	100	50***
C (Private - Capital)	5	6.4	0	0	100	100	100	0	0	0
D (Private - Capital)	9	11.5	13	14.4	100	100	100	0	0	0
E (Public - Town)	0	0	4	4.5	100	100	92	100	0	0
F (Public - Town)	0	0	12	13.3	100	100	83	100	0	0
G (Public - Town)	0	0	6	6.7	100	100	92	100	100	50***
H (Public - Town)	0	0	0	0	100	100	83	0	0	0
I (Public - Town)	0	0	0	0	100	100	100	100	100	50***
J (Public - Town)	0	0	0	0	100	100	75	0	0	0
K (Public - Town)	0	0	0	0	100	100	83	0	0	0
Total	78	100	90	100	100	100	90.9	54.5	36.3	50

Note: the maternity hospitals are named by letters to maintain the privacy of the institutions. The term “town” refers other municipalities in the State. *NICU: Neonatal Intensive Care Unit; **IMCU: Intermediate Care Unit; ***partial adherence.

Only six maternity hospitals (54.5%) performed the risk classification of pregnant women, and four (36.3%) had treatment protocols in cases of hemorrhages, although presenting partial healthcare provider adherence (Table 1).

The presence of pediatrician on call 24 hours a day and the availability of equipment for eventual maternal and neonatal resuscitation were reported as existing in all maternity hospitals(100%). However, the absence of at least one important medication was observed in 9.1% of them (Table 1). Missingdrugs were: surfactant (three units), hydralazine (twounits), methylergometrine (one unit), and silver nitrate (seven units) (datanot shown in the table).

For compliance with the ENC components, among those who received prenatal care (n=763), it was observed that 74.7% (n= 570) of the women performed six or more prenatal visits and 61.3% (n=468) received orientation on the referral services for birth during this process. The greater number of prenatal consultations shown to be associated with the use of the private service (86.1%, OR 2.29, 95%CI 1.29-4.05). Theorientation on referral services for birth was less in the public services (57.8%, OR 0.25, 95%CI 0.14-0.44) (Table 2).

**Table 2:** Proportional distribution of Essential Newborn Care components according to the type of service used by the pregnant woman in a state sample (n=768). Sergipe, Brazil, 2015-2016.

	Type of service (%)		p-value	OR (95%CI)	Total - n (%)
	Public	Private			
≥6 prenatal visits (n=763)	73	86.1	0.004	2.29 (1.29-4.05)	570 (74.7)
Prenatal orientation on referral services for childbirth (n=763)	57.8	84.3	<0.001	0.25 (0.14-0.44)	468 (61.3)
Use of corticosteroids between 26-34 weeks (n=21)	22.2	50.0	*0.905	0.90 (0.77-1.04)	5 (23.9)
Use of the partograph in labor monitoring (n=528)	40.2	25.8	0.111	1.93 (0.84-4.41)	208 (39.4)
Companion during all birth moments (n= 768)	14.1	42.1	<0.001	4.43 (2.84-6.89)	138 (18.0)
Early skin-to-skin contact (n=760)	40.8	42.6	0.719	1.07 (0.71-1.62)	315 (41.4)
Breastfeeding in the first hour of life (n=768)	37.6	5.6	<0.001	0.09 (0.04-0.22)	254 (33.1)

OR: *Odds Ratio*; 95%CI: 95% confidence interval (comparisons always between Public *versus* Private); *Fisher’s exact test.

Only 18% (n=138) of the parturients had the presence of companions during pre-birth, birth and postpartum. The highest frequency of the companion during all birth moments was observed in the private maternity hospitals (42.1%, OR 4.43, 95%CI 2.84-6.89) and the shortest frequency in maternities located outside the capital (12.2%, OR 0.47, 95%CI 0.32-0.70) (Tables 2 and 3).

**Table 3:** Proportional distribution of Essential Newborn Care components according to the place of birth in a state sample (n=768). Sergipe, Brazil, 2015-2016.

	Place of birth (%)		p-value	OR (95%CI)	Total - n (%)
	Capital	Town			
≥6 prenatal consultations (n=763)	75.7	73.9	0.581	1.09 (0.79-1.52)	570 (74.7)
Prenatal orientation on referral services for childbirth (n=763)	68.0	53.6	<0.001	0.54 (0.40-0.73)	468 (61.3)
Use of corticosteroids between 26-34 weeks (n=21)	36.4	11.1	*0.857	0.85 (0.70-1.02)	5 (23.9)
Use of the partograph in labor monitoring (n=528)	24.2	55.2	<0.001	3.86 (2.66-5.60)	208 (39.4)
Companion during all birth moments (n=768)	22.6	12.2	<0.001	0.47 (0.32-0.70)	138 (18.0)
Early skin-to-skin contact (n=760)	48.5	31.9	<0.001	2.00 (1.49-2.70)	315 (41.4)
Breastfeeding in the first hour of life (n=768)	25.5	42.3	<0.001	0.46 (0.34-0.63)	254 (33.1)

Note: the term “town” refers to non-capital state’s municipalities. OR: *Odds Ratio*; 95%CI: 95% confidence interval (comparisons always between Capital *versus* Town); *Fisher’s exact test.

The descriptive results demonstrated that the use of antenatal corticosteroids between 26 and 34 weeks was low (23.4%, n=5), mainly in maternities outside the capital (11.1%) when compared to maternities located in the capital of the State (36.4%). Partograph was used in 39.4% (n=208)of all labor monitoring (n=528); the highest percentages were observed in maternities outside thecapital (55.2%, OR 3.86, 95%CI 2.66-5.60) and inthe public services (40.2%, OR 1.93, 95%CI 0.84-4.41) (Tables 2 and 3).

After delivery, 41.4% (n=315) of the women had early skin-to-skin contact, excluding from this analysis eight women whose children received supplemental O_2_ and were admitted to the NICU, and 33.1% (n= 54) breastfed their baby in the first hour of life. The highest frequency of early skin-to-skin contact occurred in maternities located in the capital city (48.5%, OR 2.00, 95%CI 1.49-2.70). The maternal breastfeeding in the first hour of life was less frequent in private services (5.6%, OR0.09, 95%CI 0.04-0.22) and in maternities in the capital of the State (25.5%, OR 0.46, 95%CI0.34-0.63) (Tables 2 and 3).

Table 2 shows the distribution and coverage of ENC items studied in maternity hospitals in the state of Sergipe, Northeast Brazil.

## DISCUSSION

Perinatal care in Sergipe has interesting distortions. Althoughmany structures and processes are better classified than the national average, these advantages have not reflected in a better infant and neonatal mortality coefficient.^14^ Therefore, it is possible that there may be interference of other risk factors for the occurrence of these deaths in the state. A prospective cohort demonstrated that the increase in neonatal mortality in Brazilian Northeast capitals is associated with extreme low birth weight, males and Apgar with 5 minutes <7.^15^It was observed that all NICU beds and 75.5% of IMCU beds are in the state’s capital city. The maternity hospitals in Sergipe present the number and the distribution of NICU and IMCU beds according to the current legislation.^16^

It is worth mentioning that maternity hospitals outside of the capital city should care for only the common risk deliveries, since they do not have a NICU in service. The farthest maternity hospital is 118.4 km from the capital city. Thus, in order to minimize unfavorable neonatal outcomes, delivering women are recommended to receive care in services that are compatible with their gestational risk.^1^ However, this does not always occurs, and newborns who are transferred to other units after birth have five times greater chance of neonatal death.^17^ Furthermore, it should be noted that only half of the maternity hospitals studied are high risk referral maternity hospitals, which may result in delay in the transportation of the pregnant woman to the most appropriate referral service and, consequently, contribute to increase maternal and neonatal morbimortality.

A national study^13^ found that, in Northern and Northeastern Brazil, more than half of the maternity hospitals are public and located in the capital cities. In Sergipe, in turn, only two public maternity hospitals are in the capital city, while seven are in the other towns. There was no shortage of NICU and IMCU beds in Sergipe. According to the data presented in our study, the number of NICU and IMCU beds meets the Ministry of Health’s recommendation.^16^ The availability of hospital beds for newborns, as well as the provision of specialized equipment for neonatal care, is not uniform in Brazil and may explain the inequalities in neonatal mortality rates across its regions.^18^ In Sergipe, bad processes performances can be responsible for our results.

Less than 20% of public, private or mixed maternity hospitals in Brazil have NICU.^13^ Regional inequalities can be described worldwide. In Brazil, there was an increase in the number of neonatal beds, although in a fragmented way, both structurally and technically. Problems, such as the need for more physical space, more specialized human resources, and more material resources, were identified as obstacles for a complete organization of the intensive neonatal services.^19^ In Chile, there are 46 neonatal centers, eight being focused on high complexity cases. In Zambia, there is only one neonatal referral service with 25 available incubators and an occupancy rate ranging from 79 to 86 patients/day. This difference in the provision of services has a reflection in neonatal mortality rates: 10per 1,000 live births in Chile,^20^ and 36 per 1,000 live births in Zambia. In the United States, where there is a better organization and distribution of NICU beds, the neonatal mortality rate is four per 1,000 live births.^21^

Among the studied maternity units, only six performed gestational risk classification, and four had organized protocols for eclampsia, preeclampsia, and hemorrhages. In some cases, there was a partial adherence to these protocols. The prioritization of health care services, based on obstetric risk criteria, improves obstetric care and favors the humanization of the parturition process.^22^

Similar findings were described by Bittencourt et al.^23^ Theirstudy reported that 47% of the newborns with an increased obstetric risk were born in public hospitals without a NICU in Brazil, and this percentage increased to 60% in the North and Northeast regions. By our study, the presence ofa pediatrician on call during 24 hours and the availabilityof equipment for eventual maternal and neonatal resuscitation were identified in all maternity hospitals in the State. Inthese items, Sergipe state presents better results than the rest of the country and much more than other Northeast states. Accordingto Bittencourt et al.,^13^ only 37.3 % of the public institutions and 34.9% of the mixed ones presented all medicines and resuscitation materials in the North and Northeast of the country. However, this has not yet reflected in the reduction of infant and neonatal mortality in the state. The coefficients of infant/neonatal mortality in Sergipe (17.6/12.2 per 1,000 live births) are higher than in Brazil (15.3/10.6 per 1,000 live births) and similar to those in the Northeast region (18/12.7 per 1,000 live births).^14^

According to the ENC, it was observed that 74.7% of the women had performed 6 or more prenatal visits, and 61.3% had been advised on the referral service for birth during this process, especially those who used the private services (p<0.05). Similar relative frequencies were identified in a national study performed with 23,940 puerperal women, that found 73.1% of women had 6 or more prenatal visits and 58.7% had orientation on the referral service for birth.^24^There is a strong association between a greater number of prenatal visits and less perinatal deaths.^10^

Pregnant women usually search birth care service on their own, especially in risk situations, occurring sometimes a “pilgrimage” to various services that aggravates the initial risk.^2^ InSergipe, although 61.3% of the women had received orientation on the referral service for birth, a significant percentage of them still had to seek more than one service to deliver their children (29.4%). It is also believed that the main causes of this phenomenon in Sergipe are the lack of vacancies in the reference maternity for childbirth and/or the absence of any member of the multiprofessional team, as obstetricians, anesthesiologists and pediatrician/neonatologists at the time of this woman’s search for health care.^25^

Despite the benefits of antenatal corticosteroid use in reducing neonatal mortality by 40%, as well as intracranial hemorrhage by 52%, this practice has not been disseminated. Its international use ranges from 0.8 to 81%.^26^ InBrazil, the percentage ranged 50% in maternity hospital schools to 4% in public maternity hospitals.^27^ A national study has found the use of antenatal corticosteroids in only half of neonates with birthweight less than 1,000 g.^28^ Maternities of Sergipe also presented a low use of antenatal corticosteroids between 26 and 34 weeks (23.9%). A 90% coverage of ECN components is recommended in order tosignificantly reduce neonatal mortality.^2^ It is important to emphasize in relation to antenatal steroids that private and the capital’s maternity hospitals have a better performance when compared to public and countryside’s maternities, which may be related to the phenomenon of peregrination to childbirth, since the delay in reaching a more structured reference service contributes to the restriction of time for the steroid administration.

The presence of companion during all hospitalization is considered essential for a positive experience in parturition since this support brings comfort, safety, and tension relief for the parturient.^29^Only 18% of the women could have this experience in Sergipe. A similar result has been identified in the entire country (18.8%).^24^The lack of privacy in the pre-birth rooms has led many maternity hospitals to allow only female companions, thus restricting thepossibilities of choice and excluding the presence ofthe child’s father.^30^ Likewise, the absence of adequate physical space and the impossibility of maintaining the privacy of each pregnant woman during labor was pointed out by maternity hospitals’ managers as one of the main difficulties in the present study.

Partograph is an instrument that allows the diagnosis of alterations and indicates the appropriate care to correct these deviations, while still helping to avoid unnecessary interventions.^31^Its use was verified in only 39.4% of all labor monitoring in the state. The low use of partograph was also observed in a similar study, that showed this use in only 35.7% of the surviving newborns and in 36.5% in those who did not survive.^6^Another national study also observed failures in the use of the partograph, since 51% of the 428women followed by this instrument were examined less than once every two hours on media average.^32^ TheWHO recommends the partograph use during labor since 1994, because this instrument allows the early detection of labor dystonia and adequate intervention.^33^

It was also found that 41.4% of women had early skin-to-skin contact and 33.1% breastfed their babies in the first hour of life. Some possible reasons for non-breastfeeding within the first hour of life may be child health problems, mother health problems and delay in the rapid HIV test result.^34^ Thelow breastfeeding coverage in Sergipe may be attributed to the fact that this is not a very stimulated practice in the birth room, requiring the awareness of the multidisciplinary team on this aspect.^35^

The study limitations are related to the reliability of the structure data reported by the managers of the 11 maternity hospitals studied at the time of the interview, since the direct verification of the topics present in the questionnaire by the researchers was not performed. It should be noted that only the availability of the materials is insufficient to ensure thatthe health needs of women and newborns are actually met in these establishments.

The results of the present study show that, regarding the structure and work processes, including the ENC practices, the coverage of all evaluated items was considered low in Sergipe, independently of the type of hospital, public or private. Moreover, despite the adequate number of NICU beds, the presence of a pediatrician 24 hours a day and sufficient coverage of most needed medications, there is a non-compliance or a partial adherence of health professionals to both maternal and neonatal care protocols. Thus, there is an urgent need for training of medical and nursing teams to practice the evidence based protocols. Finally, we suggest the construction or use of standardized tools indicators in the maternity units to measure how much these processes are actually being implemented, as well as to give feedback to the team, aiming for a constant improvement of the quality of maternal and neonatal assistance.
